# Three Cases of Amblyopia With Fusion Maldevelopment Nystagmus Successfully Treated With Dichoptic Treatment

**DOI:** 10.7759/cureus.75496

**Published:** 2024-12-10

**Authors:** Yo Iwata, Yuki Kusayanagi

**Affiliations:** 1 School of Allied Health Sciences, Kitasato University, Sagamihara, JPN; 2 Ophthalmology Examination Department, Sakoh Eye Clinic, Kawasaki, JPN

**Keywords:** amblyopia, amblyopia treatment, dichoptic treatment, fusion maldevelopment nystagmus, latent nystagmus, occlusion therapy

## Abstract

We present three cases where traditional occlusion therapy with an eye patch proved ineffective for treating amblyopia associated with fusion maldevelopment nystagmus. In these cases, we transitioned to dichoptic therapy using the Occlu-pad, achieving successful outcomes. Eye patch occlusion of the sound eye consistently exacerbated nystagmus, whereas dichoptic treatment did not. Visual acuity improved to 0 (logMAR) in all cases following dichoptic therapy. This approach demonstrates the effectiveness of dichoptic treatment for managing amblyopia in patients with fusion maldevelopment and nystagmus.

## Introduction

Fusion maldevelopment nystagmus (FMN) is an eye movement disorder characterized by involuntary nystagmus, which results from the failure of the eyes to fuse images properly during early development. Previously known as latent nystagmus and manifest latent nystagmus, FMN is a collective term for these two types of nystagmus, which become more pronounced when one eye is occluded. The nystagmus exhibits a rapid phase toward the open-eye side, and when the occluded eye changes, the direction of the rapid phase reverses, in accordance with Alexander's law [1]. FMN is thought to result from vision deprivation during infancy, strabismus, or fusion maldevelopment caused by significant anisometropia [2]. While nystagmus intensifies when one eye is occluded, it is often present even when the binocular is open without occlusion, a condition referred to as manifest latent nystagmus [3]. It has been reported that 24% of patients with nystagmus have FMN [4].

FMN often results in amblyopia due to early-life vision deprivation, strabismus, or anisometropia. In FMN, nystagmus intensity increases under monocular conditions, which historically made occlusion therapy with eye patches contraindicated [3,5]. However, some studies suggest that full-time occlusion therapy can improve visual acuity [6] and reduce nystagmus intensity [7]. Despite this, the exacerbation of nystagmus under monocular conditions limits the effectiveness of occlusion therapy for treating FMN-related amblyopia, making management more challenging compared to amblyopia without nystagmus [8,9].

In recent years, dichoptic treatment has emerged as an alternative to traditional occlusion therapy for amblyopia [10]. This approach reduces interocular suppression and aims to improve visual acuity and stereoscopic vision by balancing visual input from each eye through interactive tasks, such as games [11]. Occlu-pad therapy enables dichoptic treatment using white-screen technology, allowing the amblyopic eye to view a normal tablet screen whereas the sound eye perceives a white screen [10]. Unlike traditional occlusion therapy, the Occlu-pad allows treatment during the binocular opening. Because it does not involve occluding one eye, it potentially prevents the nystagmus exacerbation observed in FMN during monocular occlusion. To date, no reports have specifically addressed the use of dichoptic treatment for FMN-associated amblyopia. In this study, we present three cases where patients with FMN-related amblyopia who struggled with traditional eye patch therapy achieved successful outcomes using the Occlu-pad for dichoptic treatment.

## Case presentation

Three cases of FMN were examined, as summarized in Table 1. To prevent an increase in nystagmus, a +5.00-diopter lens was used to occlude the eyes during visual acuity measurement [12]. Case 1 was diagnosed with exotropia at the age of one year, while cases 2 and 3 were diagnosed with infantile esotropia at the ages of 10 and six months, respectively. Case 1 underwent surgery for strabismus at the age of three, and case 3 underwent surgery at the age of one year and three months.

**Table 1:** Characteristics of each of the three cases. Compliance rate: (implementation time/instruction time) * 100; PD, prism diopter; DVD, dissociated vertical deviation.

Case number	1	2	3
Sex	Male	Female	Female
Fusion maldevelopment nystagmus	+	+	+
Manifest latent nystagmus	-	+	+
The age at which occlusion therapy was started	3 years, 6 months	3 years	3 years, 3 months
The time for occlusion therapy indicated	2 hours per day	2 hours per day	4 hours per day
Compliance rate of occlusion therapy (self-report)	50%	40%	20%
Compliance rate of Occlu-pad	94%	80%	82%
Ocular alignment (alternate prism cover test)	Constant exotropia; near: exotropia of 8 PD; far: exotropia of 12 PD, DVD: (+)	Constant esotropia; near: esotropia of 30 PD; far esotropia of 30 PD, DVD: (+)	Constant esotropia; near: esotropia of 8 PD; far: esotropia of 14 PD DVD: (+)
Refraction value	R: S+2.50 D C-1.00 D Axis 180°. L: S+4.50 D C-5.00 D Axis 165°	R: S+3.25 D C-0.75 D Axis 5°. L: S+2.75 D C-0.75 D Axis 10°	R: S+1.75 D C-0.75 D Axis 180°. L: S+2.00 D C-0.50 D Axis 180°
Other symptoms	Diagnosis of exotropia at 1 year old. Surgery was performed for exotropia at age 3 years old. Residual strabismus after surgery (+)	Diagnosis of infantile esotropia at 10 months old	Diagnosis of infantile esotropia at 6 months old. Residual strabismus after surgery (+). Surgery was performed for esotropia at age 1 year and 3 months old

**Figure 1:**
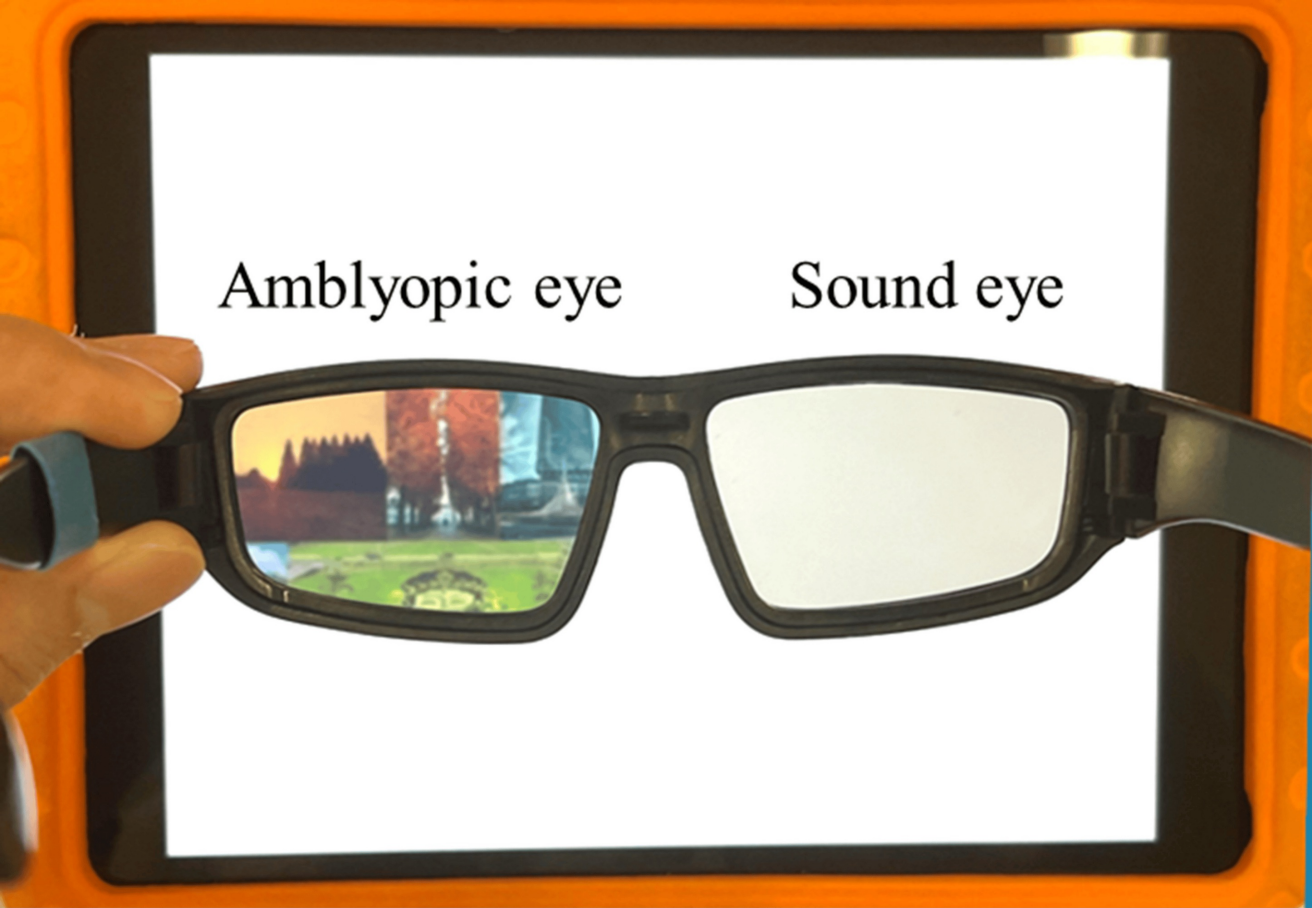
Illustration of the Occlu-pad, a device designed for dichoptic treatment. The amblyopic eye (left eye) has a clear view of the tablet screen, while the sound eye (right eye) perceives only a white screen. This image was taken and edited by the author.

**Figure 2:**
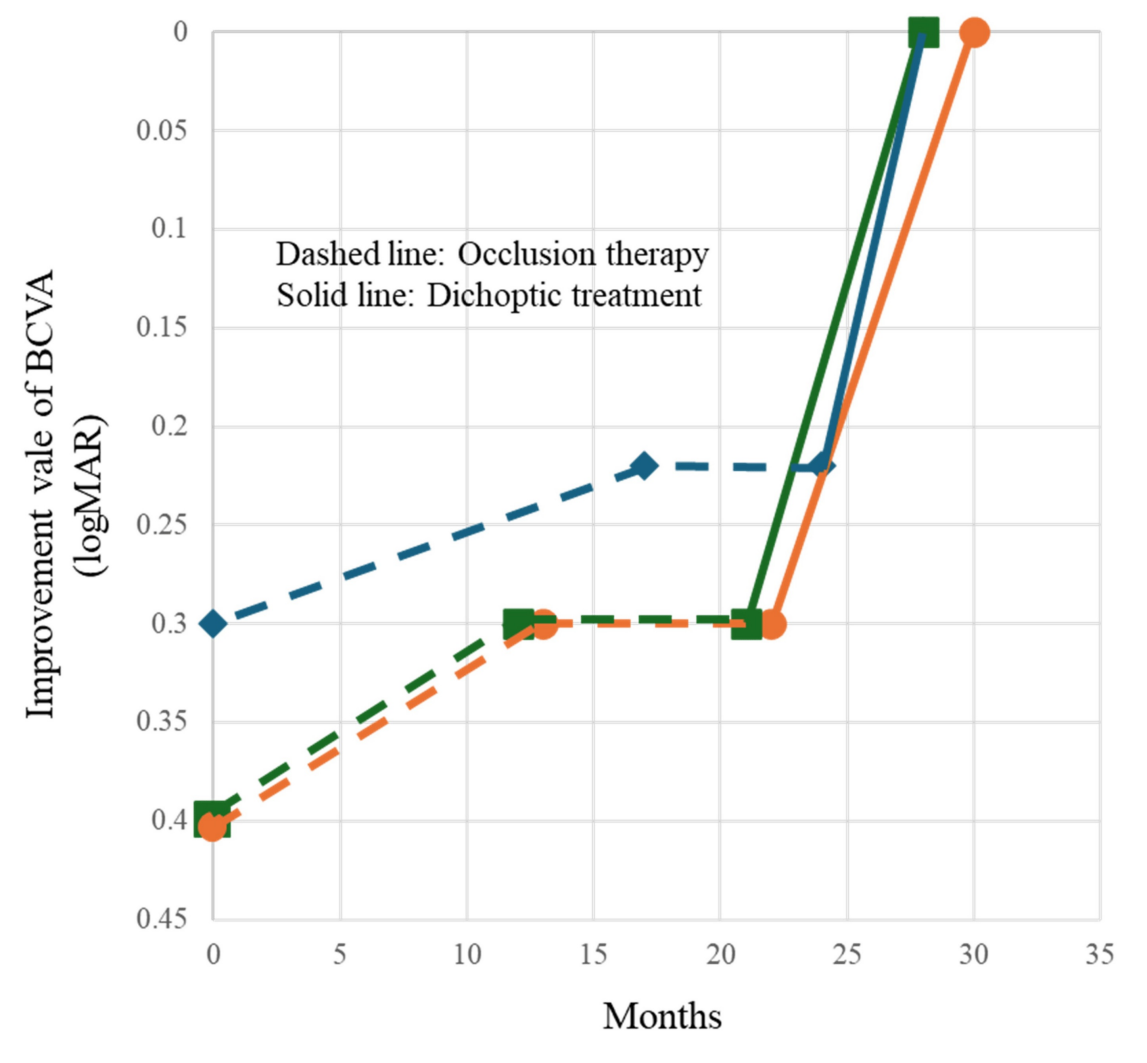
Changes in BCVA for each case. Graph depicting changes in best corrected visual acuity (BCVA) for each case. The dashed line represents treatment with occlusion therapy, while the solid line denotes the period of dichoptic treatment. Blue indicates case 1, orange indicates case 2, and green indicates case 3.

All patients presented with amblyopia. The best corrected visual acuities (BCVA) for cases 1 to 3 were 0.30, 0.40, and 0.40, respectively, based on logMAR values (Table 2). The visual acuity test was conducted at a distance of 5 m, using the Landolt ring optotype. To treat amblyopia, all patients were prescribed fully corrected glasses and instructed to perform occlusion therapy with an eye patch. The treatment commenced at three years and six months for case 1, and at three years and three months for both cases 2 and 3. Occlusion therapy schedules varied: two hours daily for cases 1 and 2, and four hours daily for case 3. However, monocular occlusion led to an increase in nystagmus across all cases. Although occlusion therapy using glasses and eye patches was performed, the improvement in BCVA was stalled. Self-reported compliance rates for occlusion therapy were 50%, 40%, and 20% for cases 1-3, respectively. The BCVA stagnation periods lasted seven months for case 1 and nine months for cases 2 and 3, with BCVA stabilizing at 0.22, 0.30, and 0.30, respectively. Therefore, occlusion therapy was discontinued, and dichoptic treatment using the Occlu-pad was initiated (Figure 1). Dichoptic treatment required twice-weekly hospital visits, with each session lasting 30 minutes. During treatment with Occlu-pad, the patient played games and watched videos. Notably, no increase in nystagmus was observed during the Occlu-pad therapy. As a result of treatment using the Occlu-pad, BCVA improved to 0 (logMAR) in all cases. The treatment periods to achieve this outcome were four months for case 1, eight months for case 2, and seven months for case 3 (Figure 2). The compliance rates for treatment using Occlu-pad were 94%, 80%, and 82% for cases 1-3, respectively. In all cases, Occlu-pad training was continued for at least three months, with the continuation of wearing glasses thereafter. One year after achieving a BCVA of 0, visual acuity was maintained in all cases.

**Table 2:** Visual acuity for each of the three cases. BCVA, best corrected visual acuity.

Case number	1	2	3
BCVA of the sound eye before and after treatment	0.00	0.00	0.00
BCVA of the amblyopic eye before initiation of occlusion therapy	0.30	0.40	0.40
BCVA of the amblyopic eye at the end of occlusion therapy	0.22	0.30	0.30
Periods with no improvement in BCVA of the amblyopic eye with occlusion therapy	7 months	9 months	9 months
BCVA of the amblyopic eye achieved with Occlu-pad	0.00	0.00	0.00
Treatment period using Occlu-pad	4 months	8 months	7 months

## Discussion

This study demonstrated that switching from occlusion therapy with an eye patch to dichoptic treatment using an Occlu-pad led to significant improvements in visual acuity in all cases, despite prior stagnation with traditional occlusion therapy. Occlusion therapy, the most widely used method for treating amblyopia, forces reliance on the amblyopic eye, often yielding positive results [13]. However, in FMN, occlusion of one eye exacerbates nystagmus [3], reducing the effectiveness of amblyopia treatment, complicating management, and prolonging the treatment period [8,9]. In contrast, dichoptic treatment provides visual stimulation to the amblyopic eye while maintaining binocular vision. Consequently, no increase in nystagmus was observed during Occlu-pad therapy in this study, and it is thought that good treatment effects were obtained.

Additionally, compliance with occlusion therapy was poor across all patients. This is thought to be that the patient is unable to be proactive about treatment because the weak eye becomes even more difficult to see due to the enhancement of nystagmus caused by occlusion of the monocular eye. Because compliance with occlusion therapy is self-reported, it is possible that it is even worse. On the other hand, adherence to dichoptic treatment using the Occlu-pad was notably better, even with the requirement of regular hospital visits. This improved compliance can likely be attributed to the absence of nystagmus exacerbation during treatment and the reduced psychological resistance, as the sound eye was not occluded.

Another treatment for amblyopia is penalization [14], which is a binocular open-type treatment that aims to improve visual acuity by applying atropine eye drops to the sound eye and creating opportunities for the use of the amblyopic eye. Although there are disadvantages to penalization, such as difficulty seeing the sound eye during treatment and side effects such as fever, it may be effective for amblyopia with FMN because it does not involve occlusion, but further investigation is needed.

Dichoptic treatment aims to eliminate interocular suppression by simultaneously engaging both eyes [10,11]. Although it is not the original aim, it is thought to be a good treatment for amblyopia with FMN. This is the first study to report a dichoptic treatment for amblyopia with FMN. However, the effects on treatment of the presence or absence of manifest latent nystagmus, history of surgery, and the direction of strabismus are still unknown. Given the limited sample size of three cases herein, it is necessary to increase the number of cases in future studies.

## Conclusions

This study is the first to report on the use of dichoptic treatment for amblyopia associated with FMN, demonstrating its effectiveness in achieving favorable outcomes. This is because dichoptic treatment does not occlude one eye; therefore, there is no increase in nystagmus, as observed during occlusion therapy. Therefore, this approach enhances patients’ acceptance and facilitates better compliance.

Given its non-occlusive nature and the absence of nystagmus exacerbation, dichoptic treatment appears to be effective in treating amblyopia with FMN.
